# Impact of plasticity and stress history on thermal volume changes in clays

**DOI:** 10.1038/s41598-025-03083-8

**Published:** 2025-05-30

**Authors:** Hamed Hoseini Mighani, Saeed Tourchi, Arash Alimardani Lavasan, Fatemehsadat Hosseini, Janos Szendefy

**Affiliations:** 1 Department of Engineering Geology and Geotechnics, Budapest University of Technology and Economics, Budapest, Hungary; 2 Computational Soil Mechanics and Foundation Engineering (COMPSOIL), Faculty of Science Technology and Medicine, University of Luxembourg, Luxembourg, Luxembourg; 3 Department of Mineralogy and Geology, University of Debrecen, Debrecen, Hungary

**Keywords:** Natural hazards, Solid Earth sciences, Engineering, Civil engineering, Energy infrastructure

## Abstract

This endeavor explores fine-grained soils’ thermally induced volumetric behavior through a series of temperature-controlled oedometer experiments under drained conditions. Undisturbed clay samples were subjected to incremental heating and cooling to evaluate the effects of over-consolidation ratio (OCR), stress history, and soil plasticity. Results revealed that normally consolidated clays undergo significant plastic contraction during heating. Over-consolidated samples showed contraction-dominated responses, highlighting the limitations of OCR as a standalone predictor of thermal behavior, with stress history emerging as a key factor. Furthermore, the influence of soil plasticity was pronounced, with high-plasticity clays experiencing greater thermal contraction due to enhanced microstructural rearrangement and mineralogical effects. The heating and cooling cycle further highlighted the irreversibility of volumetric changes in normally consolidated states, while over-consolidated samples exhibited reduced thermal hysteresis. These findings offer a detailed understanding of thermally induced volume changes in fine-grained soils, revealing the interplay between stress state, consolidation history, and intrinsic soil properties. The insights gained are foundational for advancing predictive models, optimizing the design of thermally loaded geo-energy systems, and addressing climate-driven challenges such as soil-atmosphere interactions and landslide susceptibility.

## Introduction

The interplay between thermal and mechanical loads on fine-grained soils is a subject of growing importance in geo-energy applications such as geothermal energy structures^1,2^, nuclear waste repositories^3–5^, and soil–atmosphere interactions^6–8^. These applications expose soils to complex and sustained thermal loading, necessitating a good understanding of their coupled thermal-mechanical behavior to ensure structural safety.

Fine-grained soils are especially critical in these contexts due to their physicochemical interactions^9–11^. Experimental studies have explored the behavior of various fine-grained soils during temperature changes under drained heating tests. For normally consolidated samples, the results show volumetric plastic contraction. In contrast, highly over-consolidated samples (OCR > 10) tend to exhibit thermal expansion. For soils with an intermediate OCR range (approximately 4 to 8), samples often show initial thermal expansion, which transitions to plastic contraction as temperatures increase further^12–16^. Research also suggests that the temperature at which this transition occurs increases as the OCR of the samples increases^16–21^.

Although the primary focus of this study is thermally induced volumetric behavior under drained conditions, the influence of temperature on shear strength is also relevant. The literature reports inconsistent outcomes, showing that shear strength variations with temperature depend significantly on material type, stress history, and drainage conditions^17,21,22^. Recent experimental studies indicate notable decreases in shear strength with increasing temperature in sand-clay mixtures^22,23^, reconstituted deep-water sediments^24^, and normally consolidated kaolinite clay^25^. Similarly, research on Boom clay and Opalinus clay found strength reductions and increased ductility upon heating^26^.

Despite these general trends, experimental observations have revealed some discrepancies in the soil responses. For instance, a study by Cekerevac^27^ has reported constant transition temperatures for samples with different OCRs, contradicting commonly observed trends. Similarly, Hueckel et al.^28^ observed that intact samples of Pasquasia clay (OCR = 5.7) exhibited a lower transition temperature compared to remolded samples of Pontida clay (OCR = 2.5), deviating from the anticipated behavior where transition temperature increases with OCR. Towhata^15^ and Burghignoli^29^ reported that highly over-consolidated samples (OCR>10) prepared by the reloading technique instead of the commonly unloading method did not exhibit initial expansion and thermal volume changes were dominated by contraction instead of expansion, which contradicts the results from the literature. Additionally, Abuel-Naga^21^ evaluated the engineering behavior of soft Bangkok clay under elevated temperatures, further highlighting inconsistencies across different soil types and preparation methods. These discrepancies underscore the significant influence of soil structure, inter-particle interactions, and preparation methods (recent stress history) and highlight the need for systematic investigations to clarify how natural and remolded soil behaviors diverge under thermal loads.

This study explores the thermal behavior of undisturbed fine-grained soil samples from Budapest, Hungary, with a particular emphasis on how plasticity and over-consolidation affect volumetric changes. The samples underwent a series of drained heating and cooling tests using a temperature-controlled oedometer apparatus. This setup, designed with precision and control in mind, provided the necessary thermal and mechanical control, enabling us to monitor the volumetric responses during the experiments continuously. This level of control instills confidence in the reliability of our results and the robustness of our research methodology.

## Material and methodology

**Fig. 1:**
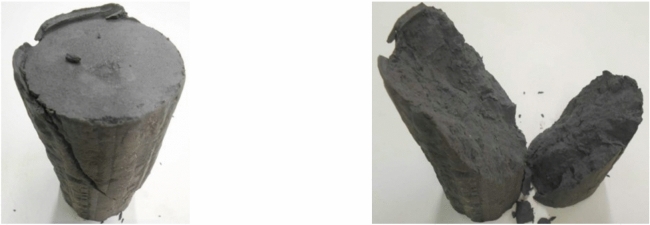
Photo of (**a**) Sample A and (**b**) Sample B.

Undisturbed soil samples were obtained from two locations in Budapest, identified as *Sample A* taken from district 13 and *Sample B* taken from district 8. In District 13, samples were taken from 13.5 m depth using a Wirth B1 drilling rig, ensuring precise extraction. The Geobor S system employed a mud flushing technique with a 100.4 mm plastic liner pipe to maintain soil integrity. In District 8, sampling at 8.5 m depth utilised a MASSENZA MI3232 drilling rig, achieving optimal recovery (Fig.  1). To minimise disturbance, a Geobor S triple-wall system extracted 100.40 mm core samples using water flushing.

**Fig. 2:**
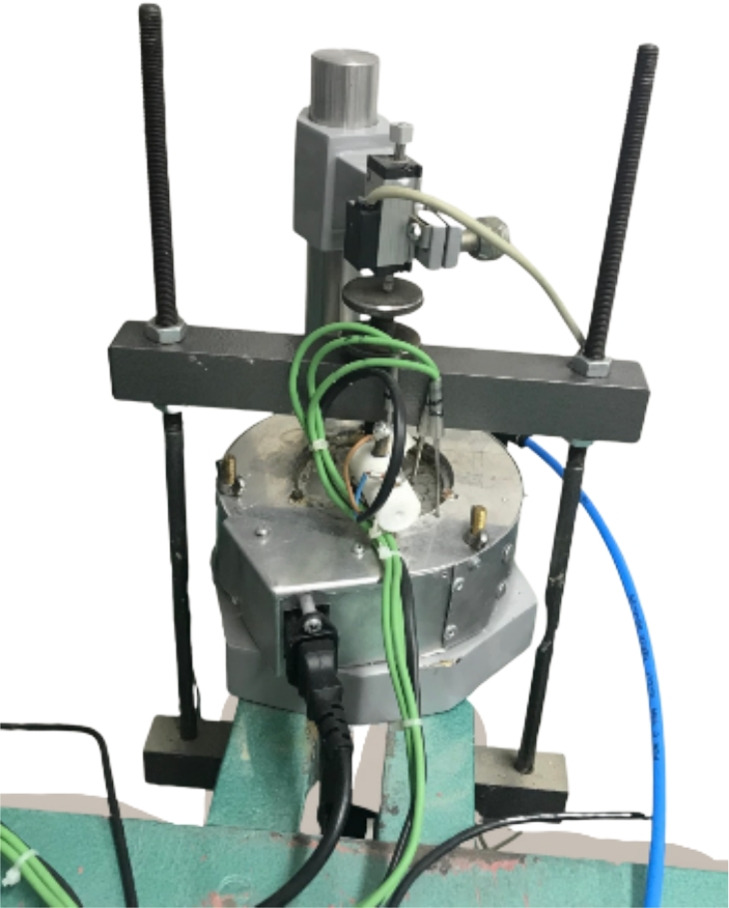
Modified thermal consolidation test device.

Soil classification and index properties were determined according to the MSZ EN ISO 14688-2:2018 standard. Sample A is a low-plasticity (LP) clay with a liquid limit of 41–$46\%$, and a plasticity index of 16–$21\%$, and Sample B is high-plasticity (HP) clay with a liquid limit of 55–$61\%$ and a plasticity index of 35–$38\%$. The experiments were conducted using a temperature-controlled oedometer apparatus. The apparatus consisted of a cylindrical copper cell designed to minimize horizontal deformation and ensure uniform heat transfer (Fig.  2).

Thermal loading was applied using an electrical heater surrounding the cell, with insulation to prevent heat loss. The thermal controller unit maintained a consistent heating rate, while a Linear Variable Differential Transformer (LVDT) measured vertical displacement with a precision of 1 $\mu$m. Temperature measurements were captured using Type K thermocouples and a data acquisition system, achieving an accuracy of $\pm \,0.1\,^\circ \hbox {C}$. To address water evaporation at elevated temperatures and ensure saturation during the tests, a water supply system was employed, including a water reservoir, an electric valve, and a water sensor to replenish lost water. To ensure drainage conditions and prevent excess pore pressure buildup, a slow temperature gradient of 0.3$\,^\circ C/hr$ was adopted during thermal loading. The soil samples were collected from below the groundwater table and considered saturated. For each test, the specimen was cut from the in-situ samples into oedometer rings with a diameter of 75 mm and a height of 20 mm, respectively.

### Drained heating tests

In the drained heating tests, samples were subjected to a stepwise mechanical loading path to establish either a normally consolidated (NC) or over-consolidated (OC) state, as summarized in Table 1. For samples LP1 and HP1, the recent stress history involved loading to their target stress levels, while for samples LP2, LP6, HP2, and HP6, the stress history included unloading from higher stress levels to achieve the desired OCR values.

For highly over-consolidated samples (LP12 and HP22), the only load applied was the weight of the loading cap (approximately 5 kPa). Given the in-situ preconsolidation pressures of 60 kPa and 110 kPa respectively for Samples A and B, the over-consolidation ratios (OCR) for these cases were calculated as 12 and 22, respectively. These details provide insight into stress histories and preparation methods employed for each sample. Following mechanical preloading, the samples underwent drained heating, with temperatures gradually increased from $25^\circ \text {C}$ to $85^\circ \text {C}$ under constant effective stress conditions.

**Table 1:** Summary of drained heating tests.

ID	Loading	OCR	Thermal Loading
LP1	5$\rightarrow$1200	1	25$\rightarrow$85
LP2	5$\rightarrow$1200$\rightarrow$600	2	25$\rightarrow$85
LP6	5$\rightarrow$1200$\rightarrow$200	6	25$\rightarrow$85
LP12	5	12	25$\rightarrow$85
HP1	5$\rightarrow$800	1	25$\rightarrow$85
HP2	5$\rightarrow$1200$\rightarrow$600	2	25$\rightarrow$85
HP6	5$\rightarrow$1200$\rightarrow$200	6	25$\rightarrow$85
HP22	5	22	25$\rightarrow$85

### Heating and cooling tests

The heating–cooling tests were conducted on samples in both normally consolidated (NC) and over-consolidated (OC) states, excluding the highly over-consolidated samples (LP12 and HP22), which underwent heating only. The samples were prepared through a mechanical preloading process to establish the desired stress histories as summarized in Table 2. Thermal cycling involved gradually heating the specimens from $25^\circ \text {C}$ to $85^\circ \text {C}$, followed by cooling back to the initial temperature under constant effective stress. These observations are integrated with the results of the drained heating tests to provide a comprehensive understanding of the thermal behaviour of the tested soils.

**Table 2:** Summary of drained heating–cooling tests.

ID	Loading	OCR	Thermal Loading
LP1	5$\rightarrow$1200	1	25$\rightarrow$85$\rightarrow$25
LP2	5$\rightarrow$1200$\rightarrow$600	2	25$\rightarrow$85$\rightarrow$25
LP6	5$\rightarrow$1200$\rightarrow$200	6	25$\rightarrow$85$\rightarrow$25
HP1	5$\rightarrow$800	1	25$\rightarrow$85$\rightarrow$25
HP2	5$\rightarrow$1200$\rightarrow$600	2	25$\rightarrow$85$\rightarrow$25
HP6	5$\rightarrow$1200$\rightarrow$200	6	25$\rightarrow$85$\rightarrow$25

## Results and discussion

### Drained heating tests

**Fig. 3:**
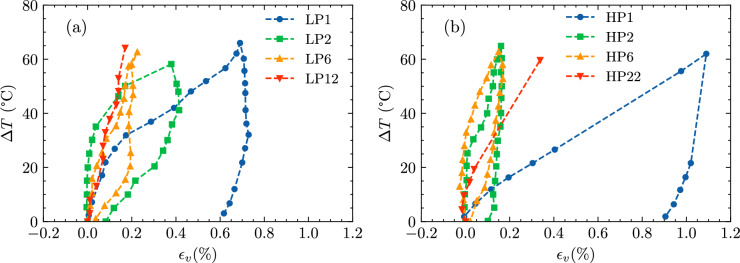
Thermal volume change after drained heating and cooling for (**a**) Low Plasticity (LP) and (**b**) High Plasticity (HP) samples, illustrating the differences in contraction and elastic behavior across OCRs.

The thermal volumetric response of selected samples under slow drained heating is examined in Fig. 3(a) and Fig. 3(b), which depict volumetric strain versus temperature increment for LP samples and HP samples, respectively. Normally, consolidated samples consistently exhibit plastic contraction during drained heating for both LP and HP samples. This behavior aligns with prior studies (e.g.,^12,27^).

Previous studies on slightly over-consolidated clay samples, including Pontida silty clay (OCR=2^30^), Kaolin clay (OCR=2^27^), and Pasquasia clay (OCR=3.4^28^), consistently exhibit purely plastic contractive thermal responses. These behaviors align closely with the observations from samples LP2 and HP2 in this study, reinforcing the understanding that clays with moderate over-consolidation ratios (OCR $\le$3.4) primarily experience volume contraction when subjected to heating.

However, all tested OC samples displayed plastic contraction—a limited initial expansion was observed for HP samples with OCR=6, which quickly diminished and transitioned to contraction. These measurements deviate from previous findings, which generally show that highly over-consolidated clay samples typically undergo an initial elastic expansion, followed by plastic contraction . This implies that OCR alone may be insufficient to predict thermal volume change.

Samples with an OCR=6 exhibited distinct thermal volumetric behaviors that were directly influenced by their plasticity levels. Specifically, the low-plasticity sample (LP6) demonstrated significant and consistent contraction during thermal loading, entirely lacking the initial elastic expansion typically expected for this OCR level. In contrast, the high-plasticity sample (HP6) showed only a minimal initial expansion before transitioning to contraction. This deviation from conventional expectations—where OCR=6 clays are generally anticipated to exhibit notable elastic expansion prior to contracting—can be attributed to the combined effects of low plasticity and the presence of sand grains within the soil matrix.

Thermal contraction in fine soils arises from a combination of interconnected mechanisms. Research indicates that under drained conditions, thermally induced pore pressure dissipation can cause volumetric contraction^18^. Elevated temperatures also reduce the thickness of the diffuse double layer, promoting particle rearrangement and volume reduce^31^. Furthermore, the decrease in viscous shear resistance of pore water at higher temperatures increases particle mobility and can trigger structural collapse^17^. In contrast, thermal expansion is partly driven by the intrinsic expansion of soil constituents^31^ and partly by enhanced inter-particle repulsion forces^32^. These forces widen inter-particle spacing and lead to the formation of larger macropores. Together, these thermal responses underscore the complex interplay between mechanical and physicochemical processes in fine soils subjected to heat. The contraction observed in low-plasticity (LP) samples, along with the absence of expansion in over-consolidated states, suggests that the thermal behavior of LP soils is largely governed by their mineral composition and low-plasticity nature. These factors limit electrochemical interactions and reduce water adsorption capacity, minimizing inter-particle repulsion. As a result, other contraction-inducing mechanisms take precedence in determining the soil’s thermal volume change.

In contrast, high-plasticity (HP) samples possess greater potential for physicochemical interaction and inter-particle repulsion. These forces intensify with rising temperature, accounting for the initial thermal expansion in over-consolidated conditions. However, at elevated temperatures, mechanisms such as double-layer compression, reduced viscous shear resistance, and pore pressure dissipation begin to dominate, leading to contraction. This may explain the relatively modest and transient thermal expansion observed in over-consolidated Sample B, especially when compared to findings reported in previous studies.

As mentioned above, the highly over-consolidated samples, namely LP12 (OCR=12) and HP22 (OCR=22), were subjected to reloading before heating. The rationale for this approach is taken from^15,29^, which investigated the effects of different stress histories—namely unloading and reloading—on the volumetric response of soils under thermal loading. Our objective in exploring the impact of stress history through various stress paths was to determine how prior mechanical loading (recent stress history) influences the thermal behavior of clayey soils. By applying unloading and reloading scenarios, we aimed to simulate real-world conditions and evaluate their impact on soil microstructure, void ratios, and volume changes during heating.

Additionally, soil plasticity emerged as a significant factor. Samples with higher plasticity (HP1 and HP22) exhibited greater plastic volumetric changes under thermal loading compared to low-plasticity samples (LP1 and LP12). The mineral composition and microstructural properties of these clays facilitate more pronounced particle movement and rearrangement during temperature fluctuations, making them more susceptible to sustained contractions.

### Heating and cooling

The thermal volume change of clay samples during heating and cooling is shown in Fig. 3(a) and Fig. 3(b) for LP and HP samples, respectively. For LP samples, the single heating and cooling cycle caused contraction across all consolidation states. The normally consolidated samples exhibited significant plastic and irreversible contraction. As the OCR increased, the extent of plastic contraction decreased notably. For highly over-consolidated samples (OCR=6), the thermal response became primarily elastic, resulting in minimal contraction during the heating–cooling cycle. This progression demonstrates the stabilizing effect of over-consolidation, which enhances the resistance of soil structure to thermally induced deformation.

HP samples—with higher plasticity—demonstrated similar trends but exhibited larger volumetric strains due to their greater thermal sensitivity. These results highlight that higher plasticity amplifies thermal sensitivity, leading to greater plastic deformation in normally consolidated states. Increasing OCR progressively reduces permanent contraction, transitioning to predominantly elastic behavior at higher OCR levels.

### Quantitative analysis of thermal volumetric response

A quantitative analysis was conducted to elucidate the relationship between volumetric strain ($\varepsilon _v$) and temperature change ($\Delta T$) in thermally loaded clays. The initial loading phase, defined by the range from zero to peak temperature increment, was analyzed using a linear relationship expressed as:

1 $$\begin{aligned} \Delta T = k \cdot \varepsilon _v\ \end{aligned}$$

where $k\,(^\circ \hbox {C}/\%)$ represents the rate of temperature increase per unit volumetric strain. This linear characterization facilitates the assessment of the initial thermal response prior to reaching the maximum temperature increment. Computed $k$ values for low-plasticity (LP) and high-plasticity (HP) clay samples, stratified by varying over-consolidation ratios (OCR), are summarized in Table 3:

**Table 3:** Quantitative parameters of thermal volumetric response for low-plasticity (LP) and high-plasticity (HP) samples, showing the slope $k$ of the linear fit $\Delta T = k \cdot \varepsilon _v$ and the semi-empirical coefficient $\alpha _T = \frac{1}{k}$.

Sample ID	OCR	$k\,(^\circ \hbox {C}/\%)$	$\alpha _T\,(\%/^\circ \hbox {C})$
LP1	1	95.65	0.01045
LP2	2	153.6	0.00651
LP6	6	278.2	0.00359
LP12	12	377.6	0.00265
HP1	1	56.88	0.01758
HP2	2	403.1	0.00248
HP6	6	418.0	0.00239
HP22	22	176.6	0.00566

Analysis of these results reveals a clear trend for LP samples, where the slope $k$ increases consistently with higher OCR. This indicates that over-consolidated LP clays exhibit a more pronounced initial thermal response, characterized by greater temperature changes for equivalent volumetric strains. Conversely, HP samples exhibit a less straightforward relationship; HP2 and HP6 samples notably display substantially elevated $k$ values compared to both normally consolidated (HP1) and highly over-consolidated (HP22) samples. Such variability underscores the intricate and nonlinear interplay between clay plasticity, consolidation history, and thermal loading.

Further investigation employed a semi-empirical approach, establishing the volumetric strain sensitivity to temperature increments through the relation:

2 $$\begin{aligned} \Delta \varepsilon _v = \alpha _T \cdot \Delta T\ \end{aligned}$$

where $\alpha _T = 1/k$ quantifies the volumetric strain per degree Celsius temperature change ($\%/^\circ \hbox {C}$). Illustrative $\alpha _T$ values are provided in Table 3. These $\alpha _T$ values reveal that normally consolidated samples (LP1, HP1) demonstrate a higher sensitivity, undergoing greater volumetric contraction per unit temperature increment. In contrast, highly over-consolidated samples (LP12, HP22) exhibit significantly lower $\alpha _T$, indicative of predominantly elastic deformation behaviors and limited plastic contraction. This observed behavior aligns well with established understandings of clay mechanics, wherein normally consolidated clays tend towards more significant plastic deformation, and over-consolidated clays exhibit largely elastic responses upon thermal loading.

## Conclusions

This study advances the understanding of thermal volumetric behavior in fine-grained soils by examining the influence of over-consolidation ratio (OCR), stress history, and soil plasticity using temperature-controlled oedometer experiments. Results demonstrate that both normally consolidated and highly over-consolidated clays predominantly undergo plastic contraction when subjected to drained heating, challenging traditional assumptions that highly over-consolidated soils expand thermally. Recent stress history significantly affects soil response; samples over-consolidated through unload-reload cycles consistently contracted upon heating, highlighting the importance of stress-path dependence. Furthermore, high-plasticity clays exhibit greater susceptibility to plastic volumetric changes compared to low-plasticity clays, underscoring plasticity as a key determinant of thermal sensitivity. These insights enhance predictive modeling and inform design considerations for geo-energy structures subjected to thermal loads.

## Data Availability

All data generated or analyzed during this study are included in this published article.
